# Family SMEs as enablers or barriers to climate neutrality: A multi-level perspective on transformational management

**DOI:** 10.1371/journal.pone.0351581

**Published:** 2026-10-14

**Authors:** Katarina Vavrová, Alexander Šarlina, Roman Lacko, Zuzana Hajduová

**Affiliations:** 1 Department of Business Finance, Faculty of Business Management, Bratislava University of Economics and Business, Bratislava, Slovak Republic; 2 Department of Management, Faculty of Business Management, Bratislava University of Economics and Business, Bratislava, Slovak Republic; 3 Department of Tourism, Faculty of Commerce, Bratislava University of Economics and Business, Bratislava, Slovak Republic; Alexandru Ioan Cuza University: Universitatea Alexandru Ioan Cuza, ROMANIA

## Abstract

**Background:**

Family firms are the dominant ownership form among European SMEs, and socioemotional wealth theory predicts their environmental behaviour in opposing directions: long-term orientation favours stewardship, capital-commitment aversion favours retrenchment. Because firm-level ownership data linked to environmental outcomes do not exist at European scale, this literature substitutes the small-firm share of a sector for the family ownership its theory concerns. We ask whether that substitution is defensible.

**Study design/methodology/approach:**

We test four hypotheses on 3,352 sector–country–year observations, twelve sectors in twenty EU member states, 2009–2023. The dependent variable is the annual decarbonisation rate: the negative first difference of log greenhouse gas intensity per unit of value added. Data come from Eurostat air emissions accounts, business statistics, environmental tax accounts and business demography, and from ECB interest rate statistics. Models use additive country, sector and year fixed effects, with errors clustered by country–sector.

**Findings/conclusions:**

No hypothesis is supported. The coefficient on SME concentration is negative throughout but insignificant (β_1_ = −0.034, p = 0.104), and the null is informative rather than inconclusive: the confidence interval [−0.074, + 0.007] excludes positive associations of consequence. Moderation by financing costs, tax exposure and enterprise maturity does not reach five per cent (p = 0.090, 0.690, 0.053). One association is robust: sectors starting from higher intensity decarbonise faster (β_2_ = +0.021, p < 0.001). A second, on tax exposure, proves an artefact of tax-base composition and of a shared denominator.

**Limitations/future research:**

Sectoral SME concentration is not a usable proxy for family-firm environmental behaviour: 58.7 per cent of its variance is sector membership, and correcting for measurement error leaves the estimate unchanged. We read this as evidence about the limits of ecological inference in family business research, not as a refutation of the theory. Progress requires linking ownership registers to environmental records.

## 1. Introduction

The European Union’s commitment to climate neutrality by 2050 has generated regulatory attention concentrated on large corporations and on installations covered by the Emissions Trading System. This orientation overlooks a structurally critical segment: small and medium-sized enterprises and, within that segment, family-owned businesses. SMEs constitute the overwhelming majority of enterprises in the EU non-financial business economy and generate more than half of total gross value added [18]; family firms represent a large share of this population [46] across manufacturing, construction, professional services and hospitality.

Despite this significance, the intersection of family business governance and climate strategy remains underexplored. Socioemotional wealth theory [1] and the resource-based view of family firm heterogeneity [7] hold that family principals derive utility from non-economic endowments, family identity, dynastic perpetuation, community standing, which generate competing expectations for environmental investment: reputational embeddedness favours stewardship, while aversion to capital commitment and control dilution favours retrenchment. Which dominates, and under what institutional conditions, has not been resolved [2], and the SME sustainability literature has largely treated ownership type as incidental [47].

The obstacle is empirical rather than theoretical. Firm-level ownership information linked to environmental outcomes does not exist at European scale, and the literature has responded by using aggregate proxies: the share of small firms in a sector, or the prevalence of family ownership inferred from survey benchmarks. This paper takes that methodological choice as its object of inquiry. We formulate the aggregate-level implications of four hypotheses drawn from the family business literature, test them on a panel assembled entirely from primary official statistics, and report what the aggregate evidence can and cannot establish.

Three research questions follow, and their order reflects the logic of the argument rather than their relative importance. First, do sectors with higher SME concentration decarbonise at systematically different rates? Second, do financing conditions, policy exposure or enterprise population maturity moderate that relationship? Third, and the question this paper is ultimately about, does sectoral SME concentration function as an informative proxy for family-firm behaviour at all? The first two are the hypotheses the family business literature has posed; the third is what their answers reveal about the research design used to pose them. The contribution is threefold: an explicit conceptual model integrating socioemotional wealth theory with the transformational management literature, with hypotheses formulated at the level at which they are tested; a panel constructed from fully documented and openly available sources spanning fifteen years; and a set of null findings that we interpret as evidence about the limits of ecological inference in family business research. Readers primarily interested in family firms should attend particularly to Sections 3.5 and 6.3, where the relationship between what the theory claims and what these data can establish is set out in full.

### Nomenclature

Table 1 lists the acronyms used in the manuscript, with expanded forms and, where applicable, units.

**Table 1:** Nomenclature: Acronyms, full names and units.

Acronym	Full name	Unit
AEA	Air Emissions Accounts (Eurostat)	–
BD	Business Demography (Eurostat)	–
CBAM	Carbon Border Adjustment Mechanism	–
CEE	Central and Eastern Europe	–
COP	Conference of the Parties (UNFCCC)	–
CSRD	Corporate Sustainability Reporting Directive	–
ETS	Emissions Trading System (EU)	–
FE	Fixed Effects (panel estimator)	–
GHG	Greenhouse gas	g CO_2_e
GVA	Gross Value Added	EUR million
MFI	Monetary Financial Institution	–
MIR	MFI Interest Rate Statistics (ECB)	% per annum
NACE	Statistical classification of economic activities in the EU	–
NFC	Non-financial corporation	–
SBS	Structural Business Statistics (Eurostat)	–
SDG	Sustainable Development Goal	–
SEW	Socioemotional Wealth	–
SME	Small and medium-sized enterprise (< 250 persons employed)	–
V4	Visegrád Four (SK, CZ, PL, HU)	–
VIF	Variance Inflation Factor	ratio

Source: The authors.

## 2. Literature review

### 2.1 Family SMEs: Structural profile and governance distinctiveness

Family businesses occupy a distinctive position because ownership, governance and management frequently overlap within one kinship network [3]. The socioemotional wealth framework holds that family principals accept below-market financial returns to protect non-economic endowments a premise substantiated across strategic risk-taking [4], R&D investment [5] and corporate social responsibility [6].

For climate strategy, the theory is internally indeterminate. Family identity is intertwined with local embeddedness and reputational standing, motivations congruent with environmental stewardship; yet preservation of socioemotional endowments counsels against the capital commitment, uncertain returns and dilution of control that characterise large-scale green investment. Evidence from Central Europe locates the difference in governance capacity rather than disposition: managerial education, formalised planning and external advisory access predict SME innovativeness [8], as does prior exposure of leadership to structured management practice [9].

### 2.2 SMEs and the climate transition: Empirical evidence

Owner-managers are typically aware of climate risk but cite financial constraints and regulatory complexity as reasons for inaction [10], and environmental engagement varies substantially across countries with sector, firm size and regulatory exposure [11]. On ownership specifically, the evidence runs against the stewardship reading. In a worldwide study of listed firms across 45 countries over eight years, family firms on average engage less in pollution prevention, green supply chain management and green product development than their non-family counterparts, a result robust to corrections for the endogeneity of family ownership [12]. A meta-analytic review of family firms and environmental performance surveys the accumulated evidence on this question [2]. The stewardship and retrenchment predictions of socioemotional wealth theory therefore, remain unresolved empirically, with the large-scale evidence currently favouring the second.

Evidence from the V4 region adds a contextual qualification: circular economy performance lags the EU-27 average, attributed to reliance on primary raw materials and assembly-type manufacturing [13,14], and countries with higher industrial intensity systematically underperform in environmental efficiency relative to GDP, with substantial within-country regional disparities [15]. SME adoption of digital tools is constrained by resource limitations rather than attitude [16], while the EU Digital Finance Strategy reshapes access to digitally intermediated capital [17].

### 2.3 The evolution of EU SME climate policy, 1993–2023

Climate policy operates on a longer horizon than any single panel window, and the position of SMEs within it has changed in kind rather than degree. Through the 1990s the Union’s environmental instruments, the eco-management and audit scheme, later recast as Regulation (EC) No 1221/2009 [20], and the IPPC Directive addressed large installations, with SMEs participating voluntarily if at all. SMEs acquired a distinct policy identity in the 2000s through the SME Definition [21] and the Small Business Act, but climate instruments remained directed at ETS installations; the Energy Efficiency Directive [22] introduced the first obligations reaching smaller firms while reporting duties stayed confined to large undertakings. The European Green Deal [23], the European Climate Law [24], the CSRD [25] and CBAM [26] then created, for the first time, a mechanism by which obligations imposed on large undertakings propagate to SME suppliers through Scope 3 reporting demands.

This periodisation matters for interpretation. The regulatory exposure of SMEs is recent, indirect and unevenly distributed across supply chains, which sets a low prior on detecting policy moderation in aggregate sectoral data over the period examined here.

### 2.4 Country groupings and shared structural characteristics

EU member states are not interchangeable observational units. Four groupings recur in the SME and family business literature, distinguished by the configuration of ownership structure, financing conditions and industrial legacy set out in Table 2.

**Table 2:** Country groupings and their shared structural characteristics.

Grouping	Member states	Shared characteristics relevant to SME decarbonisation
Nordic	SE, DK, FI	Mature governance, deep private capital markets, high policy intensity, low carbon endowment in the SME-dominated service base.
Continental / Western	DE, FR, NL, AT, BE	Large industrial base with dense SME subcontracting networks; strong intermediary institutions such as chambers and development banks.
Southern / Mediterranean	IT, ES, PT, EL	Very high micro-enterprise share, family ownership prevalent, persistent financing frictions, fragmented advisory infrastructure.
Central and Eastern (incl. V4)	SK, CZ, PL, HU, RO, BG, HR, SI	Younger governance structures, first-generation ownership still dominant, assembly-type manufacturing legacy, underdeveloped private green finance.

Source: The authors, drawing on [18] and [15].

The Central and Eastern grouping is of particular interest because three of its characteristics, first-generation ownership, assembly-type manufacturing specialisation and shallow private green finance, are precisely the moderators the framework identifies as consequential. The region therefore functions less as a residual category than as the setting in which the hypothesised mechanisms should be most visible.

### 2.5 Transformational management and family firm climate strategy

Transformational management, following Burns [29] and Bass [30], refers to processes that reshape institutional identity and capabilities rather than optimising existing routines. Applied to climate strategy, it implies a shift from compliance-oriented environmental management to proactive decarbonisation leadership, entailing reconfiguration of value chains and investment in clean technology.

The distinction is consequential for family firms specifically. A governance model emphasising continuity and stability may predispose incumbents toward incremental improvement while under-investing in transformational change. Succession transitions represent an inflexion point at which reorientation becomes more feasible, as incoming family members typically carry different risk preferences and stronger environmental socialisation [27,31]. Jayakumar et al. [45] argue that family businesses occupy a structurally advantageous position for leading sustainability transitions precisely because their planning horizon extends beyond the tenure of any individual manager, while Pollák et al. [48] find firm size to condition the adoption of digital marketing among Central European SMEs, a channel through which environmental commitment becomes visible to customers.

This literature supplies the second axis of the framework developed in Section 3. It also sharpens the measurement problem that motivates this study: transformational capacity is a property of firm governance, and no European statistical source records it, just as none records family ownership.

### 2.6 Research gaps and unresolved disagreements

Three disagreements motivate the hypotheses developed in Section 3. First, socioemotional wealth theory is indeterminate with respect to environmental investment: the stewardship and capital-commitment mechanisms predict opposite signs, and neither the meta-analytic evidence [2] nor the largest single cross-country study [12] settles which dominates or under what conditions. Second, the succession literature disputes whether generational transition operates through the preferences of incoming principals or through the governance formalisation that accompanies it [27,28]. Third, the SME policy literature disagrees on whether regulatory intensity reaches SMEs meaningfully absent direct obligations.

These disagreements share one empirical bottleneck: firm-level ownership data linked to environmental outcomes are unavailable at European scale. This study does not resolve that bottleneck. It contributes by making the aggregate-level implications explicit and testable, and by establishing empirically how much such evidence can bear.

## 3. Theoretical framework and hypotheses

Fig 1 presents the study framework. Two traditions, socioemotional wealth and transformational management, identify four firm-level mechanisms through which ownership structure is expected to shape environmental conduct. Each is translated into an aggregate counterpart observable at the sector–country–year level, and each such counterpart corresponds to one hypothesis. Institutional context enters as moderators operating on the mechanisms and directly on the outcome.

**Fig 1:**
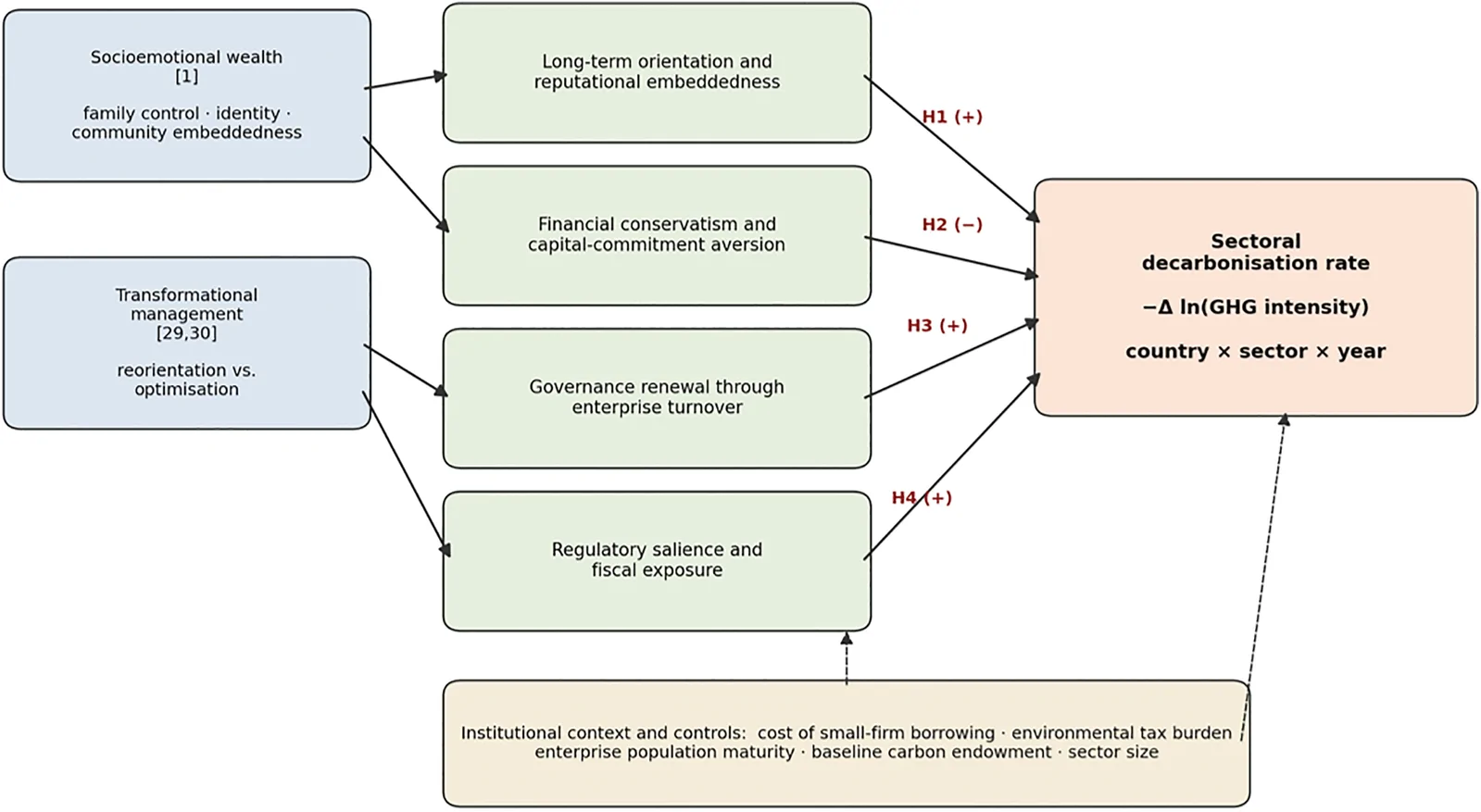
Study framework: From family-firm theory to aggregate observables. Source: the authors. Constructs in the left column are properties of individual firms and are not observed in the data; the mechanisms in the centre are their aggregate observable counterparts. Solid arrows denote hypothesised relationships tested empirically; dashed arrows denote contextual moderation. Signs in parentheses indicate the predicted direction.

### 3.1 Long-term orientation and environmental stewardship (H1)

Socioemotional wealth theory predicts a longer effective planning horizon among family principals than among professionally managed firms subject to shorter-cycle incentives [32]. A longer horizon is congruent with climate investment because material climate risks manifest over multi-decade horizons, and community embeddedness reinforces the effect through reputational channels.

**H1. Sectors with a higher concentration of SME activity exhibit higher rates of decarbonisation, other things equal.**

### 3.2 Financial conservatism and investment constraints (H2)

The same theory predicts resistance to actions threatening control dilution or committing capital under uncertainty. SMEs rely predominantly on bank credit, internal cash flow and informal family equity rather than green bonds or sustainability-linked instruments; Sedliačiková et al. [33] find for Slovak family firms that sustainable investment decisions are systematically conditioned on public co-financing availability.

**H2. The association posited in H1 is attenuated where the cost of small-firm borrowing is higher.**

### 3.3 Enterprise population maturity as a moderating factor (H3)

Generational transition constitutes a governance discontinuity capable of altering strategic orientation and firms led by later generations invest more heavily in R&D and environmental management than founder-led firms [27,28]. Since succession itself is unobservable in official statistics, we proxy the structural precondition for it: the share of the enterprise population that is established rather than newly born. Section 4.3 sets out the limitations of this proxy explicitly, and Table 3 below places it alongside the other translations the design requires.

**Table 3:** Translation of firm-level theoretical constructs into aggregate observables.

Construct (firm level)	Aggregate observable	Source	What the translation loses
Socioemotional wealth: family control, identity, community embeddedness	Share of sectoral value added produced by enterprises with fewer than 250 employees	SBS by size class [35]	Family ownership is not recorded in any European source. Size is a correlate of family control, not a measure of it; the strength of the correlation is itself unmeasured and varies by sector.
Transformational capacity: reorientation rather than optimization of routines	Share of the enterprise population that is established rather than newly born	Business demography [37]	Enterprise age is a precondition for generational transition, not evidence of it. Neither succession nor governance formalization is observed.
Financial conservatism: reliance on internal cash flow and family equity	Mean interest rate on new bank loans to firms below EUR 1 million	ECB MIR [38]	Measures the price of external credit facing small borrowers, not the propensity of family principals to avoid it. Varies by country-year only, not by sector.
Regulatory salience: perceived exposure to climate obligation	Environmental tax revenue attributed to the sector, as a share of its value added	Environmental tax accounts [36]	Captures realized fiscal incidence rather than perceived salience, and omits the supply-chain reporting channel entirely.
Environmental performance of the firm	Annual change in sectoral GHG emissions per unit of value added	Air emissions accounts [34]	Translates with little loss: both numerator and denominator are defined identically at firm and sector level and aggregate additively.
Abatement opportunity available to the firm	Log emission intensity in the entity’s first observed year	Derived from [34]	Translates with little loss, but conflates technological headroom with historical regulatory neglect.

Source: The authors.

**H3. The association posited in H1 is stronger where a larger share of the sectoral enterprise population is established.**

### 3.4 Policy exposure as an amplifying moderator (H4)

Regulatory intensity reaches SMEs principally through indirect channels: supply-chain transmission of reporting demands, and direct exposure to environmental taxation [19]. The latter is observable at the sector–country–year level and is used here as the policy intensity measure.

**H4. The association posited in H1 is stronger where sectoral environmental tax exposure is higher.**

### 3.5 From firm-level constructs to aggregate observables

Each hypothesis above is stated in terms of a construct that belongs to the individual firm and is then tested on a quantity that belongs to a sector. That translation is where most of the interpretive risk in this literature lies, and we set it out explicitly rather than leaving it implicit. Table 3 states, for every construct in the framework, the firm-level referent the theory concerns, the aggregate quantity actually observed, the source of that observation, and what the translation loses.

Two features of Table 3 govern how the results should be understood. First, no row achieves an exact translation; each loses something specific and identifiable. Second, the losses are not of the same kind. The dependent variable and the carbon and size controls translate with little distortion, because emission intensity and value added are defined identically at firm and sector level and aggregate additively. The explanatory constructs do not: family control becomes size structure, transformational capacity becomes enterprise turnover, and financial conservatism becomes a country-level price of credit. The empirical design is therefore strong on the outcome side and weak on the explanatory side, and any null result must be interpreted with that asymmetry in view.

This is not a defect peculiar to our data. It is the standard position of aggregate work in this field, ordinarily left unstated. Making it explicit converts an unexamined assumption into a testable proposition: if the translations in Table 3 are adequate, the hypotheses should find aggregate support; if they are not, the failure of the hypotheses is informative about the translations rather than about the theory. Section 5.6 supplies diagnostics bearing directly on the adequacy of these translations, and Section 6.3 returns to the disjunction once the results are known.

### 3.6 Level of analysis and the interpretation of aggregate evidence

The hypotheses are formulated at the sector–country–year level at which they are tested, not at the firm level at which the underlying theory operates. This reformulation is not cosmetic. Socioemotional wealth theory is a theory of the family principal; its aggregate implications do not follow automatically. An observed association between the SME share of a sector and that sector’s decarbonisation is consistent with the theorised firm-level mechanism, but equally consistent with compositional differences between sectors that happen to correlate with ownership structure.

Two consequences follow, and both shape how to read the results below. First, aggregate evidence can corroborate or fail to corroborate the observable implications of the theory; it cannot identify the firm-level mechanism. Second, and more restrictively, our principal explanatory variable measures the share of small and medium-sized enterprises, not the share of family-owned enterprises. No European statistical source records family ownership at sectoral granularity. The two constructs overlap substantially but are not identical, and the strength of that overlap is itself unmeasured. We therefore treat the analysis as a test of whether SME concentration functions as a usable proxy, respectively a question logically before, and answerable independently of, the family business hypotheses themselves. Throughout, we avoid causal language and state findings as associations.

## 4. Materials and methods

### 4.1 Data sources

The panel is assembled entirely from primary official statistics: Eurostat air emissions accounts [34], Structural Business Statistics by size class [35], environmental taxes by economic activity [36], business demography [37], national accounts by A*64 branch [39], and ECB MFI interest rate statistics [38]. The environmental accounts are compiled under Regulation (EU) No 691/2011 [40] and the business statistics under Regulation (EU) 2019/2152 [41]; both use the NACE Rev. 2 classification [42]. Table 4 documents each source with its dataset identifier, level of aggregation and available period, so that every series can be retrieved independently.

**Table 4:** Data sources, dataset identifiers and coverage.

Variable	Source	Dataset identifier	Aggregation	Period
Decarbonisation rate (dependent)	Eurostat, Air Emissions Accounts	env_ac_aeint_r2	Country × NACE × year	1995–2024
SME share of value added	Eurostat, Structural Business Statistics by size class	sbs_sc_ind_r2, sbs_sc_con_r2, sbs_sc_dt_r2, sbs_sc_1b_se_r2 (2005–2020); sbs_sc_ovw (2021–)	Country × NACE × size class × year	2005–2023
Environmental tax burden	Eurostat, Environmental taxes by economic activity	env_ac_taxind2	Country × NACE × year	1995–2024
Established enterprise share	Eurostat, Business Demography	bd_9bd_sz_cl_r2 (2004–2020); bd_size (2015–)	Country × NACE × year	2004–2023
Cost of small-firm borrowing	ECB, MFI Interest Rate Statistics	MIR, key M..B.A2A.A.R.0.2240..N	Country × month	2009–2023
Sectoral value added (deflation)	Eurostat, National Accounts	nama_10_a64 (B1G)	Country × A*64 × year	1975–

Source: the authors. All Eurostat series were retrieved in SDMX-CSV format from the Eurostat bulk download facility; the ECB series was retrieved from the ECB Data Portal API.

### 4.2 Sample construction and sector definitions

Emission accounts and business statistics are published in different NACE aggregations: air emissions accounts follow the A*64 branch classification used in national accounts, whereas Structural Business Statistics publishes at division level. The usable sector list is the intersection of the two. Where SBS provides finer detail than the emissions accounts, component divisions are summed to the emissions-level class; where the emissions accounts are coarser, the wider class is used. Table 5 documents every mapping decision. One mapping deserves separate note: business demography publishes fabricated metal products only in combination with basic metals (C24_C25), whereas both the emissions accounts and Structural Business Statistics provide C25 separately. The established enterprise share for this sector therefore carries contamination from a substantially more carbon-intensive activity, and Table 12 reports the model estimate without it.

**Table 5:** Sector definitions and NACE mapping between emission and business statistics.

Class	Description	Mapping
C10–C12	Food, beverages and tobacco	Aggregate: C10 + C11 + C12
C22	Rubber and plastic products	Exact
C25	Fabricated metal products	Exact (business demography only as C24_C25)
D	Electricity, gas, steam and air conditioning	Exact (section D = division 35)
F	Construction	Aggregate: whole section F
G46	Wholesale trade	Exact
G47	Retail trade	Exact
H49	Land transport	Exact
I	Accommodation and food service	Aggregate: whole section I
J62_J63	Computer programming and information services	Aggregate: J62 + J63
M69_M70	Legal, accounting, management consultancy	Aggregate: M69 + M70
N77	Rental and leasing	Exact

Source: the authors. Real estate (L68A) was excluded because emission accounts cover only 16 of the 20 member states for this class and because decarbonisation of real estate services is conceptually ambiguous.

The resulting panel covers 12 sectors in 20 member states, namely Austria, Belgium, Bulgaria, Croatia, Czechia, Denmark, Estonia, Finland, France, Germany, Hungary, Italy, Netherlands, Poland, Portugal, Romania, Slovakia, Slovenia, Spain and Sweden, over 2009–2023, yielding 3,352 complete observations across 238 country–sector entities. The panel is mildly unbalanced: some entity–year cells are absent because national statistical institutes do not publish the underlying series, and the first year of each entity is consumed by first differencing.

### 4.3 Variable construction

The decarbonisation rate is the negative first difference of the natural log of greenhouse gas emission intensity, where intensity is expressed as grams of CO_2_equivalent per euro of gross value added in chain-linked volumes with 2020 as reference. Positive values denote improvement. Differences are computed only between consecutive years, so gaps in the series do not generate spurious changes. The variable is winsorised at the 1st and 99th percentiles, affecting 70 of 3,352 observations; these originate in small sectors where a low value-added denominator produces mechanical volatility rather than genuine abatement. Table 6 sets out the construction step by step; results without winsorisation are reported in Section 5.4. One property of this construction requires flagging in advance: since intensity is a ratio, the dependent variable decomposes as the growth of value added minus the growth of emissions, so that value added enters it directly. Any regressor that is also scaled by value added therefore shares a denominator with the outcome. Section 5.6 quantifies the consequences for the environmental tax variable.

**Table 6:** Construction of the dependent variable.

Step	Operation	Detail
Step 1	Retrieve GHG emission intensity	env_ac_aeint_r2, airpol = GHG, na_item = B1G, unit = g CO_2_e per EUR (chain-linked volumes, 2020)
Step 2	Take natural logarithm	lnI_ijt_ = ln(intensity)
Step 3	First difference within entity	ΔlnI_ijt_ = lnI_ijt_ - lnI_ij_,_t_ ₋_1_, computed only for consecutive years
Step 4	Reverse sign	Decarb_ijt_ = -ΔlnI_ijt_, so that positive values denote improvement
Step 5	Winsorise	Trim at the 1st and 99th percentiles (70 of 3,352 observations)

Source: The authors.

The share of sectoral value added generated by enterprises with fewer than 250 persons employed, computed by summing the micro, small and medium size classes and dividing by the sectoral total. The micro class is taken directly where published as 0–9 and otherwise reconstructed from the 0–1 and 2–9 classes. Series before and after the 2021 methodological transition to Regulation (EU) 2019/2152 [41] are concatenated. The mean shift between 2020 and 2021 is negligible (−0.006), but its dispersion across entities is not: the entity-level standard deviation of the shift is 0.073, the range runs from −0.364 to +0.272, and 21 per cent of country–sector cells move by more than five percentage points. Year fixed effects absorb only the common component, which is close to zero here, leaving the idiosyncratic component in the error term. We therefore estimate the two subperiods separately and report them in Table 12, and we treat the difference between them as a boundary on the precision, though not the sign, of the SME coefficient.

Environmental tax revenue attributed to the sector, expressed as a percentage of sectoral value added and entered in logs. This measure is preferred to a composite policy index because it is an official statistic with a fixed definition rather than a construct with author-assigned weights, and because it varies across countries, sectors and years, so that it is not absorbed by the fixed effects.

One minus the ratio of enterprise births to the active enterprise population. This is a proxy for the structural precondition of generational transition, not a measure of succession. Enterprise age is not ownership, and business demography records neither family control nor management handover. The proxy is weak, and we treat H3 as exploratory throughout; a null result is uninformative about succession, and a non-null result would require corroboration from firm-level sources before interpretation.

The annual mean of monthly MFI interest rates on new loans to non-financial corporations up to EUR 1 million. The series covers all 20 member states from 2017 and 13 from 2009; Croatia enters in 2011 and six non-euro-area states in 2017. Rather than shortening the panel, this variable is used only in a submodel (Section 5.3) estimated on the 2,807 observations for which it is available.

### 4.4 Empirical specification

The estimating equation is:


$$\begin{array}{c} \begin{array}{c} {Decarb}_{ijt}\;=\;\alpha \;+\;\beta_{\text{1}}\;{SME}_{ijt}\;+\;\beta_{\text{2}}\;{lnI}_{\text{0}ij}\;+\;\beta_{\text{3}}\;{lnVA}_{ijt}+\;\beta_{\text{4}}\;{lnTax}_{ijt}+{\;\beta }_{\text{5}}\;{Estab}_{ijt}\;\\ +\;\beta_{\text{6}-\text{8}}\;(SME\;\times \;moderators)\;+\;\mu_{i}\;+\;\nu_{j}\;+\;\tau_{t}\;+\;\varepsilon_{ijt} \end{array} \end{array}$$

where i indexes countries, j sectors and t years; μᵢ, νⱼ and τ_t_ are additive country, sector and year fixed effects. The additive rather than interacted specification is essential. Under interacted country×sector fixed effects, any regressor that is constant within a country–sector cell, including the baseline carbon term by construction, that is perfectly collinear with the fixed effects and cannot be estimated; the design matrix is rank deficient and the affected coefficients are not identified. Under additive fixed effects, SME concentration is identified because it varies between countries within a sector and between sectors within a country, and all design matrices reported here are of full rank. Rank was verified numerically for every specification.

Standard errors are clustered at the country–sector level, giving 238 clusters. All continuous variables entering interaction terms are mean-centred, which reduces variance inflation to the range reported in Table 13 without altering the interaction estimates. Estimation is by ordinary least squares with the fixed effects entered as indicator variables.

## 5. Results

### 5.1 Descriptive statistics and correlations

Table 7 reports descriptive statistics. The mean annual decarbonisation rate is positive but modest, and its dispersion is substantial relative to its mean, indicating that year-to-year sectoral improvement is irregular rather than steady. SME concentration ranges from near zero in electricity generation to near unity in some service sectors, confirming that the variable carries genuine cross-sectional and within-entity variation.

**Table 7:** Descriptive statistics.

Variable	N	Mean	SD	Min	Median	Max
Decarbonization rate	3352	0.033	0.157	−0.492	0.031	0.567
SME share of value added	3352	0.654	0.192	0.044	0.678	1
ln baseline emission intensity	3352	4.819	1.707	0.148	4.511	10.13
ln sectoral value added	3352	8.338	1.489	3.292	8.353	12.282
Environmental taxes (% of GVA)	3352	3.895	5.956	0.024	1.319	54.777
Established enterprise share	3352	0.908	0.046	0.306	0.914	0.999
Cost of small-firm borrowing (%)	2807	3.608	1.839	1.226	3.141	13.945

Source: The authors’ computations. Decarbonisation rate is winsorised at the 1st and 99th percentiles.

Table 8 presents the correlation matrix. All pairwise correlations among the explanatory variables are well below conventional thresholds of concern, and none approaches the level at which collinearity would compromise estimation.

**Table 8:** Pearson correlation matrix.

Variable	(1)	(2)	(3)	(4)	(5)	(6)	(7)
Decarbonization rate	1.000						
SME share of value added	−0.032	1.000					
ln baseline emission intensity	−0.007	−0.410	1.000				
ln sectoral value added	0.024	−0.151	−0.152	1.000			
Environmental taxes (% of GVA)	−0.059	−0.045	0.417	−0.127	1.000		
Established enterprise share	−0.030	0.011	−0.053	0.187	−0.038	1.000	
Cost of small-firm borrowing (%)	0.005	0.045	0.137	−0.306	−0.010	−0.208	1.000

Source: the authors’ computations. Variables numbered in order of appearance in Table 7.

Table 9 profiles the twelve sectors, ordered by emission intensity. This addresses a question: which European business sectors are most directly implicated in environmental pressure, and how does SME concentration relate to that position.

**Table 9:** Sector profile: Emission intensity, decarbonisation, SME concentration and fiscal exposure.

Sector	Median GHG intensity (g CO_2_e / EUR)	Mean decarbonisation rate (% p.a.)	Mean SME share	Median environmental taxes (% of GVA)	N
Electricity, gas, steam	4,355	+3.52	0.328	3.87	283
Land transport	561	+2.32	0.613	12.03	292
Food, beverages, tobacco	282	+1.74	0.480	1.63	181
Construction	117	+0.29	0.799	7.25	293
Rental and leasing	98	+2.25	0.741	3.68	256
Rubber and plastics	78	+3.58	0.547	0.89	292
Fabricated metal products	65	+3.29	0.752	0.76	290
Wholesale trade	60	+1.77	0.783	1.18	296
Accommodation and food service	55	+3.49	0.812	1.57	295
Retail trade	48	+3.89	0.534	1.02	297
Legal, accounting, consultancy	23	+5.35	0.785	0.44	293
Computer programming, information	9	+7.68	0.609	0.25	284

Source: The authors’ computations. Sectors ordered by median emission intensity.

The spread is extreme: electricity and gas supply is roughly five hundred times more emission-intensive per euro of value added than computer programming, and land transport roughly sixty times more than retail trade. Environmental pressure among European enterprises is concentrated in four of the twelve sectors examined as electricity and gas, land transport, food processing and construction, which between them account for the overwhelming majority of the emissions attributable to this part of the business economy.

The relationship between that position and SME concentration is where the descriptive picture already anticipates the results. The two most intensive sectors have the two lowest SME shares (0.328 and 0.613), while the least intensive sectors are among the most SME-dense. Sectoral emission intensity and small-firm prevalence are therefore inversely related as a matter of industrial structure, independently of any behavioural mechanism. This is the composition effect that the fixed effects are designed to absorb and that Section 6.3 argues the SME variable continues to carry.

### 5.2 Main results

Table 10 reports four nested specifications. M1 includes SME concentration alone, M2 adds the baseline carbon term and sector size, M3 adds the environmental tax burden and the established enterprise share, and M4 adds the three interaction terms testing H3 and H4.

**Table 10:** Determinants of the sectoral decarbonisation rate, additive fixed effects.

	M1	M2	M3	M4
SME share of value added (β1)	−0.0262 (0.0234)	−0.0312 (0.0220)	−0.0269 (0.0222)	−0.0337 (0.0207)
ln baseline emission intensity (β2)	–	+0.0185*** (0.0025)	+0.0208*** (0.0025)	+0.0207*** (0.0025)
ln sectoral value added (β3)	–	+0.0143** (0.0062)	+0.0118* (0.0061)	+0.0130** (0.0060)
ln environmental tax burden (β4)	–	–	−0.0130*** (0.0032)	−0.0126*** (0.0031)
Established enterprise share (β5)	–	–	+0.0068 (0.0650)	−0.0679 (0.0738)
SME × environmental taxes (β6)	–	–	–	+0.0043 (0.0104)
SME × established enterprises (β7)	–	–	–	−0.5227* (0.2699)
SME × baseline intensity (β8)	–	–	–	+0.0079 (0.0122)
N	3352	3352	3352	3352
R²	0.0382	0.0458	0.0489	0.0503
Adj. R²	0.0251	0.0323	0.0348	0.0353
F (robust)	6.89	8.81	8.68	9.14
Clusters	238	238	238	238

Source: the authors’ computations. Standard errors clustered at the country–sector level in parentheses. All continuous variables in interaction terms are mean-centred. Country, sector and year fixed effects included in all models. *** p < 0.01, ** p < 0.05, * p < 0.10.

H1 is not supported. The coefficient on SME concentration is negative in all four specifications and does not attain conventional significance in any of them. In the fully specified model the point estimate implies that a ten-percentage-point increase in sectoral SME concentration is associated with a change in the annual decarbonisation rate of roughly two tenths of one percentage point in the direction opposite to the hypothesis, an effect that is both small and statistically indistinguishable from zero. The consistency of the negative sign across specifications is worth noting, but a consistent insignificant sign is not evidence for a reversed hypothesis.

H3 is not supported, and the point estimate carries the opposite sign. The interaction between SME concentration and the established enterprise share is negative and marginally significant at the ten per cent level in the main model (β_7_ = −0.523, p = 0.053), falling short of significance once the borrowing cost variable is included (Section 5.3). The direction contradicts the theoretical prediction. Given the acknowledged weakness of the enterprise-maturity proxy (Section 4.3), we report this as an observation warranting firm-level investigation rather than as a finding.

H4 is not supported. The interaction between SME concentration and environmental tax exposure is effectively zero (p = 0.690). Sectoral policy exposure does not moderate the relationship between SME concentration and decarbonisation in these data.

One association is robust, and a second proves not to be. The baseline carbon term is positive and highly significant in every specification: sectors starting from higher emission intensity subsequently decarbonise faster. This is a convergence pattern and is the single most stable result in the analysis. The environmental tax burden enters negatively and significantly, which should not be read as evidence that environmental taxation impedes decarbonisation. Two explanations suggest themselves, one economic and one arithmetic: the composition of the tax base, since environmental taxes fall disproportionately on energy-intensive activities whose abatement options are already substantially exploited; and the denominator that this variable shares with the dependent variable, both being scaled by value added. Section 5.6 tests both and finds the second decisive, and we withdraw the coefficient from substantive interpretation accordingly.

### 5.3 Financing conditions (H2)

Table 11 reports the submodel including the cost of small-firm borrowing, estimated on the 2,807 observations for which the ECB series is available.

**Table 11:** Submodel including the cost of small-firm borrowing.

Variable	Coefficient	Cluster SE	p
SME share of value added (β1)	−0.0324	0.0229	0.158
ln baseline emission intensity (β2)	+0.0227***	0.0030	0.000
ln sectoral value added (β3)	+0.0151**	0.0069	0.030
ln environmental tax burden (β4)	−0.0141***	0.0038	0.000
Established enterprise share (β5)	−0.0366	0.0828	0.658
Cost of borrowing (β9)	−0.0035	0.0031	0.254
SME × cost of borrowing (β10)	+0.0126*	0.0074	0.090
SME × environmental taxes (β6)	+0.0050	0.0126	0.690
SME × established enterprises (β7)	−0.5241	0.3453	0.129
SME × baseline intensity (β8)	+0.0113	0.0149	0.449

Source: The authors’ computations. N = 2807, R^2^ = 0.0565, adjusted R^2^ = 0.038, F = 7.12. Country, sector and year fixed effects included; standard errors clustered at the country–sector level. *** p < 0.01, ** p < 0.05, * p < 0.10.

H2 is not supported. The interaction between SME concentration and the cost of borrowing is positive and marginally significant at the ten per cent level (β_10_ = +0.013, p = 0.090), carrying the sign opposite to the hypothesised attenuation: the association between SME concentration and decarbonisation is, if anything, slightly less negative where borrowing is more expensive. We do not interpret a marginal coefficient with the wrong sign as support for H2, but we report rather than set aside it. Two readings are available. The borrowing cost variable is common to all sectors within a country–year and is therefore largely absorbed by the fixed effects, so the interaction may be capturing residual cross-sectional variation rather than a financing mechanism. Alternatively, sectors with high SME concentration may be less exposed to interest rate variation precisely because their investment is financed disproportionately from internal cash flow and family equity rather than from bank credit, which would make the positive sign substantively meaningful and consistent with the financing structure documented for family firms in this literature [33]. These readings cannot be separated with country-level financing data; firm-level credit records would be required. The direct effect of borrowing costs is likewise insignificant, which is unsurprising given that this variable is common to all sectors within a country-year and is therefore substantially absorbed by the fixed effects. The baseline carbon term is unchanged in this specification, the environmental tax coefficient behaves as in the main models, and the H3 interaction weakens to insignificance.

### 5.4 Robustness

Table 12 reports nine sensitivity analyses covering the treatment of outliers, the pandemic period, the level of clustering, sector aggregation choices, country composition and the two subperiods separated by the 2021 break in the Structural Business Statistics series.

**Table 12:** Robustness of the principal coefficients across alternative specifications.

Specification	N	β_1_ (H1)	p	β_7_ (H3)	p	β_2_ (carbon)
Baseline specification (M4)	3352	−0.0337	0.104	−0.5227*	0.053	+0.0207***
Without winsorisation	3352	−0.0526*	0.052	−0.5879*	0.051	+0.0203***
Excluding 2020–2021	2895	−0.0396*	0.093	−0.4973	0.119	+0.0231***
Clustering at country level	3352	−0.0337*	0.066	−0.5227*	0.071	+0.0207***
Exactly matched NACE sectors only	2006	−0.0739***	0.008	−0.4017	0.305	+0.0235***
Excluding sector C25	3062	−0.0252	0.257	−0.4615*	0.094	+0.0209***
Euro-area member states only	2329	−0.0390	0.109	−0.5726*	0.077	+0.0192***
Pre-break SBS series only (2009–2020)	2665	−0.0169	0.457	−0.3361	0.289	+0.0172***
Post-break SBS series only (2021–2023)	687	−0.1437**	0.020	−1.5566**	0.025	+0.0352***

Source: The authors’ computations. All specifications include country, sector and year fixed effects with standard errors clustered at the country–sector level unless otherwise stated. *** p < 0.01, ** p < 0.05, * p < 0.10.

The baseline carbon coefficient is positive and significant at the one per cent level in every specification, confirming the convergence result as the most durable finding. The SME coefficient remains negative throughout, but its magnitude varies by a factor of more than eight across specifications, from −0.017 to −0.144. It attains ten per cent significance in five of the nine checks, reaching one per cent in the restriction to sectors whose NACE definitions match exactly between the emission and business statistics and five per cent in the post-break subperiod. This pattern suggests that aggregation error attenuates the estimate, since precision improves markedly when definitional mismatch is removed. It does not, however, sustain a claim of a reversed H1: the baseline specification remains insignificant, the effect size stays small throughout, and significance appears only under sample restrictions that also reduce heterogeneity.

The H3 interaction retains its negative sign in all nine specifications and reaches ten per cent significance in six of them, but it falls to insignificance both when the pandemic years are excluded and when the borrowing cost variable is added, and its magnitude ranges from −0.34 to −1.56.

The two subperiod estimates warrant separate comment because they differ substantially. On the pre-break series alone, the SME coefficient is −0.017 and far from significance; on the post-break series it is −0.144 and significant at the five per cent level, and the H3 interaction is likewise several times larger. We do not read this as evidence that the relationship strengthened after 2020. The post-break subsample contains 687 observations spanning three years, one of which is a pandemic recovery year, and estimates from so short a window are neither precise nor representative. The concatenation does introduce entity-specific measurement error, and Section 5.6 quantifies its consequences: the implied attenuation is real but modest, and correcting for it does not move the coefficient towards significance. The more defensible inference is therefore that the subperiod difference reflects the imprecision of a three-year window rather than a change in the underlying relationship, and that the aggregate design estimates this quantity with limited precision at any sample length – a limitation Section 6.3 takes up (Table 13).

**Table 13:** Variance inflation factors after mean-centring of interaction terms.

Variable	VIF
SME share of value added (β1)	1.43
ln baseline emission intensity (β2)	2.18
ln sectoral value added (β3)	1.16
ln environmental tax burden (β4)	1.44
Established enterprise share (β5)	1.25
SME × environmental taxes (β6)	1.24
SME × established enterprises (β7)	1.26
SME × baseline intensity (β8)	1.89

Source: The authors’ computations. All values fall far below conventional thresholds of concern.

Multicollinearity is not a material concern in this specification. Mean-centring of the interaction terms reduces all variance inflation factors to values between 1.16 and 2.18, well within accepted bounds, and the design matrices of all reported models are of full rank.

### 5.5 Direction of association

SME concentration is a structural feature of a sector rather than an intervention with a date, and the CSRD phase-in that would supply plausible exogenous variation applies to financial years beyond this panel. What can be tested is the direction and timing of the association, which bears on the same concern.

Table 14 reports the SME coefficient at lags of one to three years, and a regression in the opposite direction in which lagged decarbonisation predicts the subsequent change in SME concentration. If the contemporaneous association reflected investment behaviour, it should persist and probably strengthen at lags, since abatement decisions take time to register in emission intensity. It does not: the coefficient is indistinguishable from zero at every lag and changes sign between the first and second. The reverse regression is also null, so reverse causation is not the explanation either.

**Table 14:** Direction and timing of the association between SME concentration and decarbonisation.

Specification	Coefficient	p	N
Contemporaneous	−0.0269	0.225	3352
Lagged one year	−0.0043	0.860	3087
Lagged two years	+0.0167	0.457	2832
Lagged three years	+0.0046	0.851	2589
Reverse: lagged decarbonization → Δ SME share	+0.0075	0.384	3087

Source: The authors’ computations. All specifications include country, sector and year fixed effects and the full control set, with standard errors clustered at the country–sector level.

A purely contemporaneous association that vanishes at one year is difficult to reconcile with a behavioural mechanism and easy to reconcile with a compositional one, in which SME concentration and emission intensity move together within a sector-year because both respond to the same structural conditions. We report this as evidence on the nature of the association rather than as an identification strategy, and the causal language is avoided throughout.

### 5.6 Diagnostics: What the SME variable measures, and what the null can exclude

A null result carries weight only if the design could have detected the effect it fails to find, and only if the explanatory variable measures what it is asked to measure. This section reports four diagnostics addressing both requirements.

#### Where the variation in SME concentration comes from.

Table 15 decomposes the variance of the SME share. Sector membership alone accounts for 58.7 per cent of it, country for 5.3 per cent and year for 0.3 per cent. The variable is therefore, to a first approximation, an indicator of industrial composition rather than of anything that differs between countries within a given sector. After all three sets of fixed effects are removed, 35.0 per cent of the original variance remains, and that residual is the whole of the variation identifying β_1_.

**Table 15:** Variance decomposition of the SME share of sectoral value added.

Variance attributed to	R^2^
Sector	0.587
Country	0.053
Year	0.003
Sector + country	0.647
All three fixed effects	0.650
Residual variation identifying β_1_	0.350

Source: the authors’ computations. Entries are the R^2^ of a regression of the SME share on the indicated fixed effects.

This bears on the proxy question posed in Section 3.6, though it does not settle it alone. Family ownership is itself sector-structured, more prevalent in hospitality and food processing than in electricity generation, so a high sector share in the SME variable does not by itself establish that the proxy fails. What it does establish is the ceiling: any ownership-related signal must reside in the 35 per cent of variance surviving the fixed effects, since the remainder is absorbed by the same effects that would absorb a sectoral ownership pattern. The proxy question is therefore decided not here but by whether that residual variation behaves as conduct or as composition, which is what the timing tests of Section 5.5 and the translation-precision pattern of Section 6.3 address.

#### Measurement error and attenuation.

The concatenation of Structural Business Statistics series across the 2021 methodological transition introduces entity-specific error, and we initially supposed this might attenuate the estimate sufficiently to explain the null. It does not. Treating the 2020–2021 shift as the difference of two independent measurement errors, a conservative assumption, since any genuine structural change in the shift is thereby counted as noise and the attenuation correspondingly overstated, what implies an error standard deviation of 0.051 against identifying variation of 0.114, a reliability ratio of 0.796. The implied attenuation factor is 1.26: the error-corrected coefficient is −0.042 rather than −0.034, and remains far from significance. Measurement error is present and worth reporting, but it is not what produces the null.

#### Statistical power.

Table 16 reports the minimum detectable effect. At conventional 80 per cent power the design can detect coefficients of 0.058 or larger in absolute value, corresponding to a change of 0.58 percentage points in the annual decarbonisation rate for a ten-percentage-point increase in SME concentration, is roughly one sixth of the mean rate observed in the sample. The design is therefore adequately powered against effects of moderate size and underpowered against small ones.

**Table 16:** Statistical power and precision of the estimate for H1.

Quantity	Value
Estimated coefficient β_1_	−0.0337
Cluster-robust standard error	0.0207
95% confidence interval	[-0.0743, +0.0069]
Minimum detectable effect (80% power, α = 0.05)	0.0580
MDE as change in annual rate per 10 pp SME share	0.58 pp
Mean annual decarbonisation rate in sample	3.31%
MDE relative to mean rate	17.5%
Reliability ratio of SME share	0.796
Attenuation-corrected β_1_	−0.0423

Source: the authors’ computations. The minimum detectable effect is calculated as (z_0.975_ + z_0.8_) × SE.

The confidence interval is more informative than the point estimate, and it is asymmetric in a way that matters for the hypothesis. In the fully specified model, it runs from −0.074 to +0.007, and in the specification without interaction terms, where the coefficient is unconditional rather than evaluated at mean moderator values, from −0.074 to +0.012. H1 predicted a positive association, and on either reading, the interval excludes positive effects above roughly 0.01, a change of about a tenth of a percentage point in the annual rate per ten percentage points of SME concentration, or under four per cent of the mean rate. The evidence against H1 is therefore not merely an absence of support: within the precision of this design, the hypothesised direction can be ruled out. In the negative direction, the test is weaker, and effects as large as −0.074 cannot be excluded, a limitation we state plainly rather than convert into a claim of a reversed relationship.

Environmental tax burden correlates with baseline emission intensity at +0.50 and with contemporaneous intensity at +0.54, and the median burden rises monotonically across quartiles of carbon position from 0.66 to 5.71 per cent of value added, a factor of nearly nine (Table 17). Table 18 then shows that when contemporaneous emission intensity is added to the specification, the tax coefficient collapses from −0.0126 to −0.0009 and loses all significance, while remaining significant under alternative controls that do not capture carbon position directly.

**Table 17:** Environmental tax burden by quartile of baseline emission intensity.

Quartile of baseline emission intensity	Median environmental taxes (% of GVA)	Median GHG intensity (g CO_2_e / EUR)
Q1 (lowest)	0.66	17
Q2	1.16	54
Q3	1.46	121
Q4 (highest)	5.71	758

Source: The authors’ computations.

**Table 18:** Sensitivity of the Environmental tax coefficient to controls for carbon position.

Specification	β_4_ (tax)	p	β_1_ (SME)	p
Baseline (M4)	−0.0126***	0.000	−0.0337	0.104
Plus tax × baseline carbon interaction	−0.0143***	0.000	−0.0343*	0.097
Plus contemporaneous emission intensity	−0.0009	0.769	−0.0223	0.253
Plus sector × year fixed effects	−0.0096***	0.002	−0.0390**	0.047

Source: The authors’ computations. All specifications retain country, sector and year fixed effects with standard errors clustered at the country–sector level. *** p < 0.01, ** p < 0.05, * p < 0.10.

Composition is not the whole explanation, however, and a second mechanism is arithmetic rather than economic. The dependent variable decomposes as Δln(value added) – Δln(emissions), and the tax variable is tax revenue divided by value added. Value added therefore appears in the denominator of both, so that a sector-year in which value added grows will mechanically display both faster measured decarbonisation and lower measured tax burden, independently of any behaviour. Table 19 isolates this channel; all four rows are estimated on the 3,087 observations for which the growth of value added is defined, so the first row differs slightly from Table 12. Controlling for the growth of value added reduces the tax coefficient from −0.014 to −0.011; replacing the dependent variable with the change in absolute emissions, which removes value added from the denominator entirely, reduces it to −0.001 and eliminates its significance.

**Table 19:** The shared-denominator channel: sensitivity of the tax coefficient to the construction of the dependent variable.

Specification	β_4_ (tax)	β_1_ (SME)	β_2_ (baseline carbon)	N
Baseline, restricted sample (intensity denominator)	−0.0142***	−0.0404*	+0.0190***	3087
Plus control for Δln value added	−0.0114***	−0.0330	+0.0182***	3087
Dependent variable = -Δln absolute emissions	−0.0014	−0.0192	+0.0155***	3087
Restricted sample without winsorisation	−0.0173***	−0.0620*	+0.0174***	3087

Source: the authors’ computations. All specifications include country, sector and year fixed effects and the full control set, with standard errors clustered at the country–sector level. N is 3,087 rather than 3,352 throughout this table because every specification requires the growth of value added between consecutive years, which is undefined in the first observed year of each entity; the first row is accordingly the baseline model re-estimated on that restricted sample, and the small differences from Table 12 are due to sample composition alone. *** p < 0.01, ** p < 0.05, * p < 0.10.

We therefore withdraw any substantive interpretation of the environmental tax coefficient. It reflects a combination of tax-base composition and a shared denominator between regressor and outcome, and it should not be read as evidence about the effect of environmental taxation on abatement in either direction. The two conclusions that matter for this paper are unaffected: the baseline carbon term retains its sign, magnitude and significance under every construction of the dependent variable, and the SME coefficient remains negative and insignificant throughout. Reporting this diagnostic rather than the original compositional account alone is, in our view, the more useful contribution, since intensity-based dependent variables and ratio-based regressors are ubiquitous in this literature and the arithmetic applies wherever they share a denominator.

## 6. Discussion

The analysis returns null results on all four hypotheses. The discussion is organised in five parts: what the one robust association and the one artefactual coefficient indicate about country and sector patterns; how the findings relate to the international climate framework and to the financing, market and digital channels through which SMEs are reached; what the null results do and do not license; what follows for policy and for research design; and the limitations that bound all of the above.

### 6.1 Country and sector patterns of decarbonisation

The strongest result is a convergence pattern: sectors starting from higher emission intensity decarbonise faster. This is consistent with a technological interpretation in which abatement opportunities are most abundant and least costly where efficiency is initially lowest, and with a regulatory interpretation in which the most intensive activities attract the earliest and strongest policy attention. The two interpretations are not separable in these data.

The negative coefficient on environmental tax exposure is not interpreted substantively. Section 5.6 shows it to arise from two sources, neither behavioural: the composition of the environmental tax base, which concentrates incidence in activities whose energy use is structurally embedded in production technology and whose remaining abatement slack is smallest; and a shared denominator, since value added appears in both the tax ratio and the emission intensity from which the dependent variable is differenced. Removing value added from the denominator of the outcome eliminates the coefficient. We record it as a methodological caution rather than a finding.

The country groupings set out in Section 2.4 predict a gradient in which mature governance and accessible capital allow long-term orientation to translate into investment. The results do not support this expectation: restricting the sample to euro-area member states, which differ systematically from the non-euro group in financing architecture, leaves the SME coefficient essentially unchanged. Similarly, the sectoral expectation that SME-dense manufacturing would display distinctive behaviour relative to SME-dense services is not borne out; the restriction to exactly-matched sectors changes the magnitude of the SME coefficient but leaves the substantive conclusion intact.

The most informative sectoral result is negative in a different sense. If SME concentration captured a coherent behavioural disposition, its estimated association should be reasonably stable across sector definitions. Instead, it moves from −0.017 to −0.144 depending on which sectors are included, which years are used and how the classes are aggregated – an eightfold range – while remaining insignificant in the baseline specification. Variability of that kind is what one expects when a variable is proxying for sectoral composition rather than for firm behaviour.

### 6.2 International frameworks and the financing, market and digital channels

The sectoral and regional patterns connect to the wider international agenda at three points. SDG 9 on industry, innovation and infrastructure and SDG 12 on responsible consumption and production both presuppose the diffusion of production-process change through enterprise populations that are, in the European case, overwhelmingly small and family-owned [43]. The diffusion channel therefore runs precisely through the governance structures this study cannot observe, which is a limitation of the present analysis and of the monitoring frameworks attached to those goals: neither the SDG indicator set nor European business statistics disaggregate environmental performance by ownership form.

SDG 13 on climate action is pursued in the EU primarily through instruments calibrated to large emitters, leaving a coverage gap that the CSRD supply-chain cascade closes only partially and indirectly. Our convergence result speaks to this gap obliquely: aggregate improvement is concentrated where initial intensity was highest, which is broadly where ETS-covered installations are located, and the absence of any detectable SME-concentration effect is consistent with the transition being driven by regulated large emitters rather than by the diffuse enterprise population beneath them.

Successive Conferences of the Parties have shifted emphasis from national inventory targets toward implementation and finance, and the first global stocktake concluded under the Paris Agreement placed implementation capacity at the centre of the agenda [44]. The constraint identified at firm level in the family business literature and the constraint identified at international level in climate finance negotiations are arguably the same constraint observed at different scales: the absence of intermediating instruments capable of converting long-horizon environmental disposition into financed investment at small scale. Our null results neither confirm nor refute that reading, but they do establish that it cannot be tested with the aggregate statistics currently published.

### 6.3 Hypotheses results

The first is that socioemotional wealth theory is wrong about environmental behaviour. We do not endorse this reading. The theory makes claims about family principals, and our data contain no information about family ownership; a null at the sectoral level cannot refute a firm-level proposition.

The second is that the competing mechanisms within the theory offset one another, so that stewardship and capital-commitment aversion cancel in aggregate. This is consistent with our results, and it is worth noting that the largest firm-level study available finds family firms engaging less in environmental practices rather than more [12] so an aggregate null could reflect a genuine negative firm-level effect diluted by sectors in which family control is rare. The reading is not identified by our data, however: an aggregate null is equally consistent with offsetting mechanisms and with no mechanism at all.

The third is that the hypothesised mechanisms are real at firm level but too small, relative to the structural determinants of sectoral emission intensity, to register in aggregate data. This reading is compatible with the power analysis: the design detects coefficients of 0.058 or larger, and firm-level effects diluted across a sector whose members are mostly not family-controlled could fall well below that threshold. It implies that the aggregate design is underpowered by construction rather than misspecified, and that larger samples would eventually recover the effect.

The fourth, which the diagnostics of Section 5.6 allow us to adjudicate, is that the translations set out in Table 3 are inadequate, that the aggregate observables do not carry the firm-level constructs they were asked to carry. This reading is distinguishable from the preceding three because it makes predictions they do not, and three diagnostics bear on it. We state at the outset that six constructs do not constitute a test; what follows is a pattern consistent with the reading, offered as such.

The first prediction is that the problem should be composition rather than noise. The variance decomposition confirms this directly: 58.7 per cent of the variation in SME concentration is sector membership, against 5.3 per cent country and 0.3 per cent year. A variable of which six-tenths is industrial classification cannot carry much information about ownership-related conduct, whatever remains in the residual. The second prediction is that measurement error should not be the explanation, since classical noise attenuates towards zero without producing the pattern of instability observed here. This too is confirmed, and confirming it required us to abandon an interpretation we had initially favoured: the reliability ratio is 0.796 and the error-corrected coefficient is −0.042, still far from significance. The third prediction is that estimates should track the precision of the translation rather than the quantity of data. The SME coefficient is closest to significance in exactly the specification that removes definitional mismatch between the emission and business classifications, and its magnitude varies by nearly an order of magnitude across restrictions that change what the variable measures rather than how many observations it rests on.

The fourth reading is the one we regard as best supported, and it holds that sectoral SME concentration does not function as a usable proxy for family-firm environmental behaviour. The second reading remains compatible with the evidence and we do not exclude it; the first and third are less so, the first because a sectoral null cannot bear on a firm-level proposition and the third because underpowering alone would not produce estimates that are precise where the translation is clean and unstable where it is not.

The implication is not that the family business literature has been asking the wrong questions. It is that the answers it has extracted from aggregate data are underdetermined by those data. Table 3 makes the point precisely: of the six constructs in the framework, the two that translate cleanly into aggregate observables are the outcome and the abatement-opportunity control, and those are exactly the two that produce robust, well-determined coefficients here. The four that translate poorly produce, between them, three nulls and one coefficient that proved on inspection to be an artefact of shared denominators rather than a finding. Not one yielded an interpretable estimate. That correspondence between translation quality and estimation success is the strongest single piece of evidence this paper offers, and it distinguishes the fourth reading from the third: a design merely underpowered would produce imprecise estimates across the board, not precise ones exactly where the translation is clean and unusable ones exactly where it is not. If this reading is correct, the accumulation of further aggregate studies will not settle the disagreements identified in Section 2.6, and the methodological implication for the literature is substantial.

### 6.4 Implications for policy and for research design

The policy implications we draw are correspondingly narrow, and we distinguish them explicitly from broader reflection. What the evidence supports is that decarbonisation effort is concentrated where initial intensity is highest and that environmental tax exposure marks structural position rather than behavioural responsiveness. It does not support conclusions about how policy should be differentiated by firm size or ownership, because the data cannot distinguish those categories.

Beyond the evidence, but consistent with the wider literature, three channels merit attention. Where private green finance is thin, national promotional banks and guarantee institutions are the binding intermediaries, and Sedliačiková et al. [33] find that sustainable investment by Slovak family firms is conditioned on public co-financing availability. Internationalisation operates as an informal channel of environmental upgrading independent of domestic regulation, since supplying international customers makes certification a market access condition. And digital capability conditions both the reputational channel through which environmental commitment becomes visible and the financial channel through which it becomes fundable [16,17]. We present these as reflections, not as findings.

For research design, the implication is more definite. Resolving whether family ownership shapes environmental behaviour in Europe requires linking administrative ownership registers to environmental records at firm level. The staggered implementation of the CSRD offers the most promising source of quasi-experimental variation for such work. Aggregate sectoral analysis, including this one, cannot substitute.

### 6.5 Limitations

Five limitations bound the conclusions. First and most fundamentally, the principal explanatory variable measures SME concentration, not family ownership; no European source records the latter at sectoral granularity, and the overlap between the two is unmeasured. Second, the analysis is aggregate and the ecological inference problem set out in Section 3.6 applies throughout: sectoral associations do not identify firm-level mechanisms. Third, the succession proxy is weak, measuring enterprise population maturity rather than generational transition, and H3 should be read as exploratory. Fourth, six of the twelve sectors are wider aggregates than the classes originally intended, and the fabricated metal products class is available in business demography only in combination with basic metals; the corresponding robustness checks address but do not eliminate this. Fifth, the estimates are associations rather than causal effects, the identification strategy exploits no exogenous variation, and the concatenation of Structural Business Statistics series across the 2021 methodological transition introduces entity-specific measurement error that year fixed effects do not absorb; Section 5.6 shows the resulting attenuation to be modest, but the design remains underpowered against small effects, being able to detect coefficients of 0.058 or larger while the point estimate is −0.034.

## 7. Conclusion

In this paper, we asked whether sectoral concentrations of small and medium-sized enterprises are associated with the pace of decarbonisation in the European Union, and whether financing conditions, policy exposure and enterprise population maturity moderate that relationship. Across a panel of sector–country–year observations spanning fifteen years and assembled entirely from primary official statistics, none of the four hypotheses derived from the family business literature is supported.

The constraints this reveals are as much epistemic as economic. The decisive obstacle to understanding how ownership structure shapes environmental conduct in Europe is not the absence of theory, of which there is a great deal, nor the absence of data, of which there is more than the literature has used. It is the absence of a link between the two: ownership is recorded in national registers, environmental performance in statistical accounts, and the two are not joined. Every aggregate study, including this one, works around that gap rather than through it.

The challenges that follow for policy design are correspondingly ones of information before they are ones of instrument. Reporting obligations transmitted through supply chains impose informational demands on firms that often lack the internal capacity to meet them; support architectures that treat SMEs as an undifferentiated category cannot target the governance heterogeneity that determines whether support is absorbed or unused; and neither problem can be diagnosed with data that do not distinguish ownership forms. The convergence pattern we document means faster improvement where intensity is initially highest and suggests that the aggregate transition is proceeding, but says nothing about which firms bear its costs.

The prospects are nonetheless real. Generational transition is proceeding across the European family business population and brings governance formalisation with it. Supply-chain transmission of reporting obligations, whatever its short-run burden, is building the first information infrastructure through which SME emissions become visible and therefore addressable. And the staggered implementation of the CSRD will generate variation that firm-level research can exploit. The decisive question for the next programming period is whether the registers being built for compliance purposes are made available for analysis, or whether the gap this paper has documented is reproduced in a new generation of data.

Family SMEs cannot be treated as passive recipients of policy impulses transmitted from large-firm supply chains. But establishing what they actually do requires observing them, and at present European statistics do not. That is the finding we are most confident in reporting.

## Supporting information

S1 Table Data panel. (XLSX)

S1 File Data set family. (ZIP)
